# Adsorption of Amlodipine at the Surface of Tosyl─Carbon Nanoparticles for Electrochemical Sensing

**Published:** 2016

**Authors:** Mandana Amiri, Hamideh Imanzade

**Affiliations:** a *Department of Chemistry, University of Mohaghegh Ardabili, Ardabil, Iran. *; b *Department of Chemistry, Payame Noor University, Ardabil, Iran.*

**Keywords:** Amlodipine, Carbon nanoparticles, Voltammetric sensor, Analysis

## Abstract

The adsorption processes of amlodipine onto hydrophilic carbon nanoparticles (Emperor 2000^TM^) are investigated. The significant increase in voltammetric responses for pre-adsorbed amlodipine compared with those for solution confirms high affinity of amlodipine to carbon nanoparticles (possibly due to π-π stacking interaction between aromatic rings of amlodipine and surface-sulfonated carbon nanoparticles). To obtain the optimum of adsorption conditions, the effects of pH, agitation rate, and adsorption time are investigated. Under differential pulse voltammetry conditions, the peak current for the oxidation of amlodipine shows two linear relationships with concentration in the range from 1000 μM to 10.0 μM and 10.0 μM to 10.0 nM. The limit of detection is estimated to be 1.0 nM. Determination of amlodipine in real samples such as human serum and commercial tablets is demonstrated.

## Introduction

Recently, carbon nanostructures have been employed in many applications such as sensing (1). Various types of carbon nano-materials such as nanotubes (2), graphenes (3), nanoparticles (4) and nanofibers (5) have been found wide interests because of their unique physical and chemical properties which can provide an important and feasible platform for electroanalysis particularly in the design of modified electrodes for electrochemical sensing (6). 

Commercial carbon blacks, carbon nanoparticles (CNPs), are 1 to 50 nm in diameter with a high surface area accessible for chemical functionalization and ideal for effective interaction with redox active species (7). Although these materials are structurally less well-defined in comparison to carbon nano-tubes or graphene materials, they offer many opportunities for new devices and technologies, for example, nano-carbon-based sensors (8, 9). CNPs, generally exhibit extremely high surface areas, high conductivity, and a multitude of reactive surface and adsorption sites (10). CNPs may be considered more economical than other nano-carbons, as they are produced commercially in bulk quantities and also often formed as waste by-products during the formation of other nano-materials such as CNTs. Recently, The CNPs modified electrode has been applied for the determination of alizarin (11), dopamine (12), dihydroxybenzene isomers (13), acetaminophen, phenylephrine and dextromethorphane (14), tramadol (15) and naltrexone (16) with high sensitivity. 

The adsorption process of organic molecules on the carbon nano-materials has been extensively investigated. Often, the adsorption phenomena have been explained for π-π interactions (17), hydrogen bonds (18) and based on electrostatic interaction (19). The π-π interaction occurs between the organic molecule and the aromatic rings of carbon nanomaterial, while hydrogen bonds and electrostatic interaction needed the involvement of oxygen-containing groups on carbon nano-materials surface (20).

**Scheme 1:**
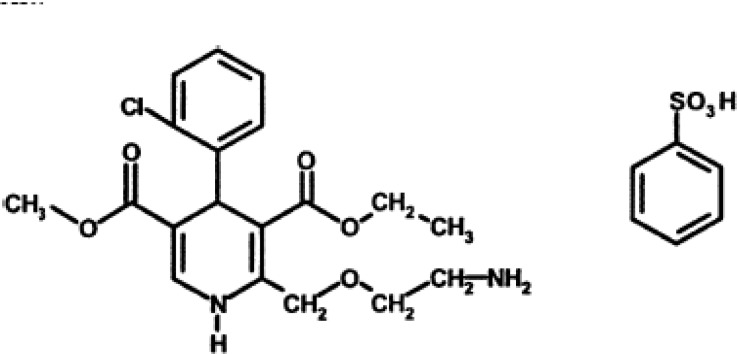
Chemical structure of Amlodipine maleate

Amlodipine (See Scheme 1.), a third-generation dihydropyridine calcium antagonist, is prescribed for the treatment of angina and hypertension. A lot of methods have been reported for the determination of amlodipine alone or in combination with other active pharmaceutical agents in dosage forms or in biological fluids (21). Various analytical methods have been reported for the assay of amlodipine besylate in pure form as well as in pharmaceutical formulations. They include high performance liquid chromatography (22–26), reversed phase high performance liquid chromatography (27–29), high performance thin layer chromatography (30, 31), gas chromatography (32), gas chromatography–mass spectrometry (33), liquid chromatography with tandem mass spectrometry (34) and fluorimetry (35).

In this paper, tosyl-carbon nanoparticles have been used. The adsorption behavior of amlodipine on the CNPs surface has been studied by electrochemical methods. It is confirmed by cyclic voltammograms (CV) that amlodipine is effectively adsorbed on CNPs surface. Based on the voltammetric studies the possible adsorption mechanism is also discussed. Due to the strong adsorption of amlodipine on CNPs modified electrode, a sensitive differential pulse voltammetric method is proposed for the determination of amlodipine.

**Table 1:** Comparison of Some Electrochemical Methods Which Previously Used for the Determination of amlodipine

**Electrode**	**LOD (M)**	**DLR (M)**	**method**	**References**
GC MWNT/EPPGE SWNT/EPPGE	1.4 × 10^−8^ 5.0×10^−9^ 1.0×10^−9^	4.0 × 10^−8^ to 2.0 × 10^−6^ 5.0×10^−9^ to 1.0×10^−6^ 5.0×10^−9^ to 1.0×10^−6^	DPV SWV SWV	[35] [37] [37]
CPE Au/o-MWCNT GC GC	2×10^–10^ ^-^ 8.01×10^-7^ 8.53×10^-7^	9.9×10^–9 ^to 1.4×10^–7^ 7.05×10^-6 ^to 2.02×10^-5^ 4.0×10^-6^ to 1.0×10^-4^ 4.0×10^-6^ to 1.0×10^-4^	DPV CV DPV OSW	[38] [39] [40] [40]
GC/CNP electrode	1.0×10^-9^	1.0×10^−8^ to 1.0×10^−5^ and 1.0×10^−5^ to 1.0×10^−3^	DPV	This work

**Figure 1:**
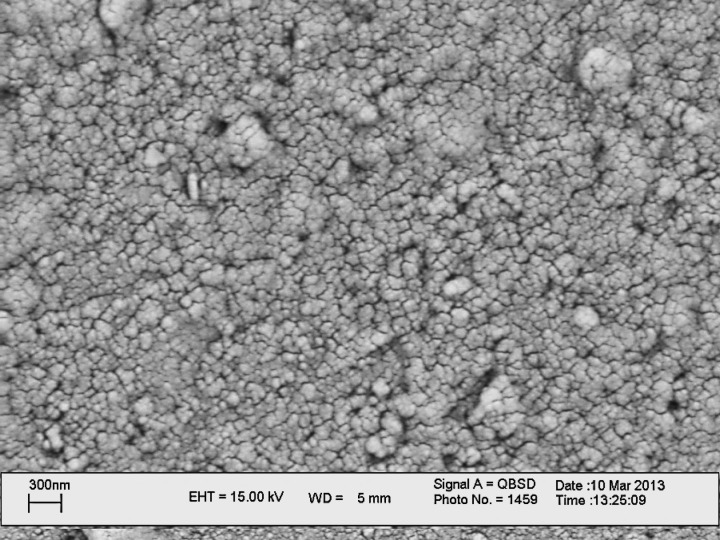
SEM image of carbon nanoparticles deposited at the surface of glassy carbon electrode

**Figure 2:**
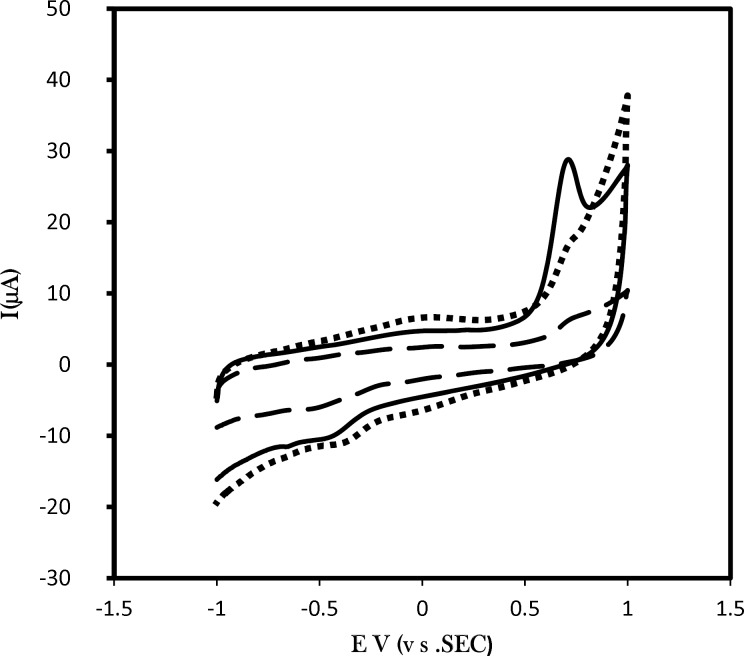
Cyclic voltammograms of 0.01 mM solution of amlodipine in buffer solution pH 7.0 (dotted line) at GC/CNPs electrode. For pre-adsorption measurements the modified electrode is immersed for 15 min in amlodipine solution and then rinsed and transfer into clean phosphate buffer solution pH 7.0 for cyclic voltammetry analysis at GC/CNPs electrode (solid line) and at GC electrode (dash-dotted). Scan rate was 100 mV s^-1^

**Figure 3:**
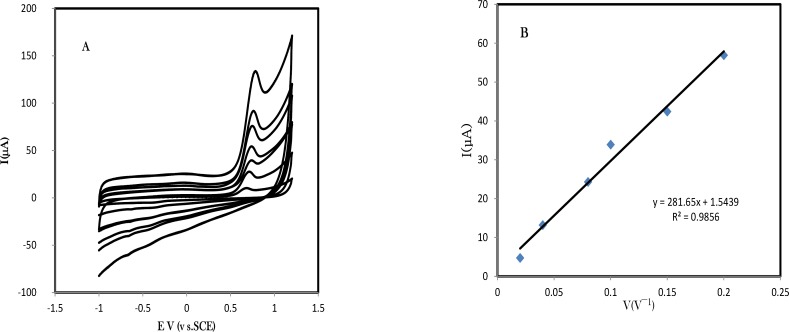
A) Cyclic voltammograms of pre-adsorbed amlodipine in various scan rates (B) Plot of the anodic and the cathodic peak current versus the scan rate consistent with an pre-adsorbed amlodipine, the modified electrode is immersed for 15 min in amlodipine solution and then rinsed and transfer into clean phosphate buffer solution pH 7.0. Scan rate was 100 mV s^-1^

**Figure 4:**
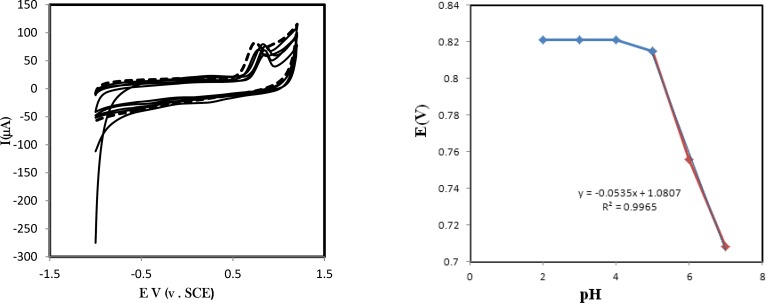
(A) Cyclic voltammograms of pre-adsorbed amlodipine at the surface of GC/CNPs in voltammetric cell buffer solutions pHs 2, 3, 4, 5, 6 and 7. Scan rate is 100 mV/s. (B) pH dependency of oxidation potential of amlodipine

**Figure 5:**
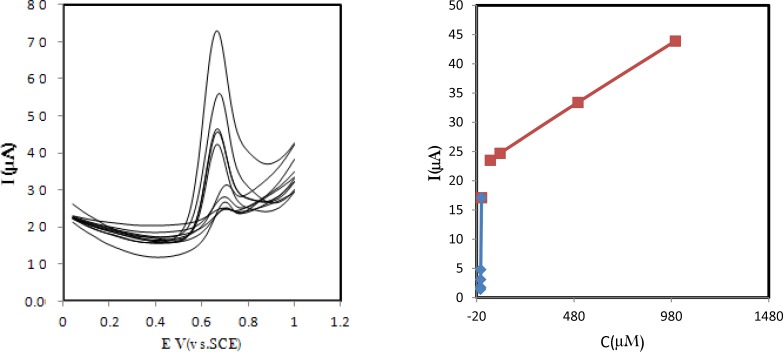
(A) Differential pulse voltammograms for the modified electrode is immersed for 15 min in various concentrations of amlodipine solution and then rinsed and transfer into phosphate buffer of pH 7.0 at the surface of GC/CNPs; Scan rate 50 mV s^−1^; pulse amplitude 50 mV; step potential 5 mV; Modulation time= 25 mV. (B) Calibration curve (dependency between concentration of amlodipine and oxidation peak current

## Experimental

*Apparatus*

Voltammetric experiments were performed using a Metrohm Computrace Voltammetric Analyzer model 797 VA. A conventional three-electrode system was used with a glassy carbon disk electrode (2 mm diameter GCE), a KCl- saturated calomel reference electrode (SCE), and a Pt wire as the counter electrode. A digital pH/mV/Ion meter (Metrohm) was applied for the preparation of the buffer solutions, which were used as the supporting electrolyte in voltammetric experiments. The scanning electron microscope (SEM) images were obtained using LEO 1430VP. 

*Reagents*

Carbon nanoparticles with surface sulfonate groups were obtained from Cabot (ca. 9 to 18 nm diameters, Emperor 2000^TM^, Cabot Corporation). Amlodipine reference was kindly provided by Sobhan Daru pharmaceutical company (Tehran, Iran). All other chemicals were analytical reagent grade from Merck. All aqueous solutions were prepared with doubly distilled deionized water.

*Procedure I: Preparation of Modified Electrodes*

Pretreatment of glassy carbon electrode (GC) was done using 2500 emery (MATADOR, Germany) rinsed thoroughly with water. A stable suspension of CNP containing 1 mg/mL in acetonitrile using 30 min ultrasonic agitation was prepared. 6 μL of this suspension was casted on the pretreated GCE surface and dried in the air to evaporate solvent. The obtained modiﬁed electrode (GC/CNPs) was characterized by scanning electron microscopy (SEM), and cyclic voltammetry techniques (CV).

*Procedure II: Adsorption of amlodipine from aqueous media onto GC/CNPs*

In the first part, 1×10^-5 ^mol L^-1^ stock standard solution of amlodipine was prepared in acetate buffer. The adsorption process was performed onto modified electrode during 15 min under stirring. Following the adsorption time, the stirrer was stopped. The electrode was taken out of the adsorption cell and was then transfer into voltammogram cell. Voltammogram was recorded by applying negative going potential from -1.0 to +1.0 V.

## Results and discussion

*Scanning Electron Microscopic study of GC/CNPs electrode*

To study the surface morphology of the CNP thin films, scanning electron microscopy (SEM) has been utilized. Scanning electron micrographs (see Figure 1.) of the carbon nanoparticle material confirm a particle size in the order of 40-100 nm radiuses. It seems the CNPs aggregate during the preparation of suspension and deposition. The thickness of the film can be estimated as approximately 300 nm. Due to the porous nature of this film water and electrolyte can readily access the film whereas electrical contact and conductivity via carbon nanoparticles is maintained.

*Voltammetric studies of adsorbed amlodipine and amlodipine solution at the surface of bare and modified glassy carbon electrode*

The affinity of amlodipine to adsorb CNPs film at the glassy carbon electrode has been studied (i) in aqueous solution of amlodipine and (ii) in pure electrolyte after pre-immersion of the electrode into amlodipine. Figure 2. shows a typical set of voltammograms for the oxidation of 6.0 µM solution of amlodipine in phosphate buffer solution pH 7.0 (dotted line) at GC/CNPs electrode. For pre-adsorption measurements the modified electrode is immersed for 15 min in amlodipine solution and then rinsed and transferred into clean phosphate buffer solution pH 7.0 for cyclic voltammetry analysis at GC/CNPs electrode (solid line) and at GC electrode (dashed line). 

The increase in the capacitive current is observed, but also the faradaic current responses are substantially increased at GC/CNPs. As can be seen, a considerable enhancement in the peak currentusing the modified electrode was obtained.

The comparison of the charge under the voltammetric peak and peak current of amlodipine in solution and adsorbed amlodipine confirms that the affinity of amlodipine to adsorb to CNP is clearly evident. This is likely to be associated with the π-π stacking interaction between aromatic rings of amlodipine and carbon nanoparticles. 

Compared with the unmodified electrode and in addition to the fact that the porous interfacial layer of the CNPs modified electrode with a high specific surface area increases the conductive area, molecules can penetrate through the conductive porous channels onto the electrode more easily, leading to higher sensitivity and selectivity. On the other hand, on the surface of the modified electrode, there are energy rich electrons in CNPs which could form π-π bonds with amlodipine molecules, consequently the electron transfer of amlodipine at the surface of the modified electrode is facilitated and exhibits catalytic effects toward the oxidation of amlodipine, it is concluded to a significantly increasing in peak current.

*The optimization of pre- adsorption of amlodipine at GC/CNPs*

To obtain the optimum of adsorption conditions of amlodipine, the effect of several parameters has been investigated at oxidation peak current of amlodipine. The time, pH and stirring rate in adsorption process were optimized. Various times 2, 5, 15, 20 and 30 min were studied and the maximum peak current has been observed for 15 min. By increasing time, adsorption process was progressing. After 15 min, all binding sites have been filled by amlodipine. An adsorption time of 15 min was used for further study. Agitation rates 200, 400, and 800 rpm were examined, the best signal was observed for 400 rpm. The role of pH in the adsorption step was investigated. The best pH for adsorption was obtained in 0.1 M phosphate buffer pH 7.0. 

*Voltammetric results of adsorbed amlodipine at the surface of GC/CNPs electrode*

The cyclic voltammetric studies for adsorbed amlodipine in optimized conditions were performed at the surface of the GC/CNPs in a buffered solution of pH 7.0 at different potential sweep rates. Figure 3A. exhibits the cyclic voltammograms of adsorbed amlodipine at the surface of GC/CNPs with various scan rates, *ν*, in the range of 10–200 mVs^−1^ in the potential range -0.1 to -0.3 V. Experiments at various scan rates indicate fast electron conduction within the CNPs film. The anodic peak current is approximately linearly related to the scan rate which confirms the amlodipine oxidation follows a surface controlled mechanism (see Figure 3B.). The resulted equations for anodic peak current versus scan rates are *I*_pa_* (μA) = 281.657 u ( Vs*^-1^*) +1.5439 (R*^2 ^*= 0.9856)*, respectively.

The linear relation between peak potential and logarithm of scan rate can be expressed as *E*p (V) = 0.825 + 0.089 log *v* (V s^-1^); *R*^2^ = 0.9941. As for an irreversible electrode process, according to Laviron, Ep is defined by the following equation:

Ep=E0+2.303 RT1-αnFlogRTK01-αnF+2.303RT1-αnFlog(vVs)

(3)

where, *α* is the transfer coefficient, *k*^0^ the standard heterogeneous rate constant of the reaction, *n* the number of electrons transferred , *ν* the scan rate and *E*^0’^ is the formal redox potential. Other symbols have their usual meanings. Thus the value of *αn* can be easily calculated from the slope of *E*p versus log *ν*. In this system, the slope was 0.089, taking *T* = 298 K, *R* = 8.314 J K^-1^ mol^-1^ and *F* = 96480 C, αn was calculated to be 0.89. Generally for an irreversible process, *α* was assumed to be 0.5. Further, the number of electron (*n*) transferred in the electro-oxidation of amlodipine in rate determining step was calculated to be ~ 1. 

From the charge under the voltammetric signal the number of binding sites can be assessed. Varying the concentration of amlodipine during the adsorption process allows the affinity or binding constant of the film to be assessed. For a Langmurian adsorption process the inverse of the charge should be linearly related to the inverse concentration that linear behavior with a slope consistent with a binding constant of 7.5×10^4^ M^-1 ^is observed.

Cyclic voltammograms of pre-adsorbed amlodipine at the surface of GC/CNPs in voltammetric cell buffer solutions 2.0 and 7.0 have been shown in Figure 4A. As can be seen, no pH dependency has been observed in pHs between 2-5. Following the peak potentials of oxidation of amlodipine, in pH higher than 5 shows a negative shift by increasing of the pH of the buffer solution. This confirms that H^+^ participates in oxidation of amlodipine. With considering the resulted slope for peak potentials, it confirms the number of electron and protons are equal in oxidation of amlodipine.

*Analytical measurement*

*Analytical Results I.: Test Samples*

The differential pulse voltammetry (DPV) technique was applied for quantitative determination of amlodipine (See Figure 5A.). The linear range for amlodipine determination was evaluated. Under the experimental condition by use of differential pulse voltammetry technique, the peak current of amlodipine had linear relationship with amlodipine concentration in the range of 1000 μM to 10.0 μM, the linear equation is *I*/µA = 20.587 + 0.024 *C/µM* (*R*^2^ = 0.9535, *C* is in M) and in the range of 10.0 μM to 0.01 μM, the linear equation is *I*/µA = 1.5193 + 3.122 *C/µM * (*R*^2^ = 0.9998, *C* is in M) (See Figure 5 B.). Limit of detection was estimated 1.0 nM based on ten measurements. Table 1. compares some electrochemical methods which exist in literature. The presented modified electrode is less expensive most of the electrode. In addition, it exhibits the better analytical results.

*Analytical Results II.: Real Samples*

For calculating the applicability of the proposed route in the real sample analysis, it was used to determine amlodipine in human serum and commercial tablets. The compound was determined in pharmaceutical tablet sample containing appropriate amount of compound by using standard addition method. Tablet samples were powdered and an aliquot was prepared in 0.1 M phosphate buffer with pH 7.0. The slope of the calibration curve, which is obtained by the spiked standard solutions of amlodipine in the range of 1000 μM to 10.0 μM, was 0.023 A/M a correlation coefficient of (R^2^) 0.9535, the recovery of 95.0% was calculated.

Besides, Recovery tests of amlodipine were carried out by spiking of compounds in human serum. The slope of the calibration curve, which was obtained with the spiked standard solution of amlodipine in the range of 10.0 μM to 0.01 μM, was 3.122 μA/μM with a correlation coefficient of (*R*^2^) 0.9998. Compared with the standard curve, 3.2175 μA/μM a recovery of 97.1% was obtained with the new method, revealing that the method is appropriate for accurate determination of amlodipine in real and complex human serum samples.

*Reproducibility of sensor preparation*

The reproducibility of the modified electrode was investigated in the presence of two concentration levels (high and low) of amlodipine in buffer solution pH 7.0 and potential scan rate 0.1 V s^−1^ by using voltammetric measurements for eight measurements. The relative standard deviations for amlodipine determination, based on the eight replicates of analysis were 4.8% for 1× 10^−4^ M and 4.9% for 5×10^−7^ M.

## Conclusion

It has been shown that carbon nanoparticles provide excellent substrate materials for oxidation of amlodipine. A Langmuir-type binding isotherm was observed with binding constant of 7.5×10^4 ^M^-1^ and two linear amlodipine detection ranges of 1000 μM to 10.0 μM and 10.0 μM to 10.0 nM. Determination of amlodipine in real sample has been demonstrated.
